# Impact of the COVID-19 pandemic on teaching and learning in health professional education: a mixed methods study protocol

**DOI:** 10.1186/s12909-021-02871-w

**Published:** 2021-08-19

**Authors:** Arunaz Kumar, Mahbub Sarkar, Elizabeth Davis, Julia Morphet, Stephen Maloney, Dragan Ilic, Claire Palermo

**Affiliations:** 1 Faculty of Medicine, Nursing and Health Sciences, Monash University, Melbourne, Australia; 2 Department of Obstetrics and Gynaecology, Faculty of Medicine, Nursing and Health Sciences, Monash University, 246 Clayton Road, Victoria 3168 Clayton, Australia; 3 Faculty of Medicine, Nursing and Health Sciences, Monash University, Peninsula campus, Franskton, Victoria Australia

**Keywords:** Distance education, Face-to-face, Remote learning, Graduate outcome, Readiness, Online curriculum, Evaluation, Work-based, E-learning

## Abstract

**Background:**

Due to the complex nature of healthcare professionals’ roles and responsibilities, the education of this workforce is multifaceted and challenging. It relies on various sources of learning from teachers, peers, patients and may focus on Work Integrated Learning (WIL). The COVID-19 pandemic has impacted many of these learning opportunities especially those in large groups or involving in person interaction with peers and patients. Much of the curriculum has been adapted to an online format, the long-term consequence of which is yet to be recognized. The changed format is likely to impact learning pedagogy effecting both students and teachers. This requires a systematic approach to evaluation of online teaching and learning adaptation, in comparison to the previous format, where, in person education may have been the focus.

**Methods:**

The proposed study is a broad based evaluation of health professional education in a major Australian University. The protocol describes a mixed methods convergent design to evaluate the impact of online education on students and teachers in health professional courses including Medicine, Nursing, Allied Health and Biomedical Science. A framework, developed at the university, using Contribution Analysis (CA), will guide the evaluation. Quantitative data relating to student performance, student evaluation of units, quantity of teaching activities and resource utilization will be collected and subjected to relevant statistical analysis. Data will be collected through surveys (500 students and 100 teachers), focus groups (10 groups of students) and interviews of students and teachers (50 students beyond graduation and 25 teachers, for long term follow up to 12 months). Application of CA will be used to answer the key research questions on the short term and long-term impact of online education on teaching and learning approaches.

**Discussion:**

The protocol describes the study, which will be widely implemented over the various courses in Health Professional Education and Biomedical Science. It will evaluate how students and teachers engage with the online delivery of the curriculum, student performance, and resources used to implement these changes. It also aims to evaluate longitudinal outcome of student learning attributes and impact on graduate outcomes, which is poorly reported in educational literature.

## Background

The preparation of the future healthcare workforce is a key priority for governments around the world[1]. Traditionally this education has mainly been supported through a variety of campus-focused activities, which involve face-to-face interactions of students with peers, and work-integrated learning (WIL). For decades, doctors, nurses and allied health professionals have been trained by observing and learning from experienced clinical practitioners through work-integrated learning, similar to the “apprenticeship model”[2]. Student learning typically takes place in locations like lecture theatres, hospital wards, operating theatres, practitioners’ clinics and the community. Occasionally it occurs informally in social places like hospital tearooms, cafeterias or in workplace corridors through tutor-student and peer interactions, but is typically in person[3].

In person education has been shown to create more student-tutor and student-student interaction, which can promote better engagement [4]. As explained through the Social learning Theory, new patterns of behavior arise from a direct interaction with peers or by observing behavior of peers[5]. However, due to the COVID-19 pandemic all in person opportunities for formal and informal learning have ceased and health professional courses have been required to move to exclusive delivery through online education[6]. The impact of this change to teaching and learning, on both learners and teachers, is largely unknown.

Online education is the delivery of learning materials using internet for student-student and student-teacher interaction and for distributing educational materials. Over the last decade, with advancement of technology-assisted learning, teachers have started using online learning platforms to promote self-directed learning and assessment in students. Use of online education also assists in engaging a large group of students at one time (where lectures may not be possible) with options of both synchronous and asynchronous learning [7]. While synchronous learning ensures that all students learn the same content in a similar way, asynchronous learning facilitates information to be communicated across sites and campuses, with students engaging in learning at their own pace, and where feasible in their own time. Similarly, online delivery of education as a component of blended learning, allows tutors to flexibly adjust to student learning styles and assess them [8]. It may assist teachers to provide the necessary support required for the individual student while feedback may not be possible in a large group face-to-face session [9].

During the COVID-19 pandemic, universities across the world have transitioned to distance education, most of which, is planned for online delivery [6, 10, 11]. Health professional courses may use variable tools of blended learning for this process, which may include synchronous online tutorials, E-learning in simulation sessions, asynchronous activity in moderated discussion forums, formative quizzes and other teacher-directed or self-directed learning activities. Engaging with these learning methods may be perceived differently from conventional classroom-based teaching. Online learning has required adjustment by both teachers and learners to adapt to new learning styles with focus on active learning and technological support required for delivery of teaching [12, 13].

Engaging with a curriculum that has been transitioned from in person to online is likely to impact how students learn and how they can contextualize that learning into clinical practice. Understanding the impact of these initiatives on student engagement, learning and behavior (both positive and negative), will provide important information for teaching and learning practice into the future[14], in particular the influence of online education on the development of practical skills and graduate readiness to practice. In this paper, we present a protocol developed in order to study the process and impact of student and teacher adaptation to the learning pedagogies developing as a result of the pandemic. The primary aim of this protocol is to study the impact of the change to teaching and learning approaches. This includes evaluation of the online education and changes to work-integrated learning, during the COVID-19 pandemic and its impact on students and teachers, both in the short and long term.

## Methods/Design

The study is based at the Faculty of Medicine, Nursing and Health Sciences (FMNHS) at Monash University, a large research-intensive university offering medicine, nursing and 12 other health professions and five health science courses. The study will have student and teacher participation from the various courses across the faculty.

### Participants

Participation will be sought from different health professions. A purposive sample of courses was chosen to provide rich insights. Courses were chosen based on being those that are most affected by WIL (or not) for students and for being larger in size. The health professional courses included are Doctor of Medicine (MD 5-year course), Bachelor of Nursing (3-year course), Bachelor of Physiotherapy (4-year course), Bachelor of Health Science (3-year course –WIL elective) and Bachelor of Biomedical Science (3-year course – WIL elective only). Students will be recruited from all years of their respective courses. All teachers (academics, clinical educators, affiliate staff) working at the faculty contributing to the above courses will be invited to participate in the study. Both student and teacher participants will be offered to participate in the study on a voluntary basis. The total number of participants is anticipated to be 500 students and 100 teachers, focus groups (10 groups with 4–8 students per group) and individual interviews of students (50 students for long term follow up to and beyond graduation) and teachers (25 teachers) for a 12-month period.

### Study Duration

The study is proposed over 12 months commencing May 2020 till May 2021 and for students to be followed up to and beyond graduation in 2021. The study duration has been proposed based on the assumption that students are likely to return to in person teaching (partly with the exception of large group teaching) at the beginning of 2021 in Australian universities. The re-introduction of face-to-face teaching is likely to occur in stages. The evaluation instruments are designed to capture various data collection points over the timeframe that occur during and also following the time, when students and teachers use online education.

### Ethics

Ethical approval for this study has been obtained from the Monash University Human Research Ethics Committee (approval number 24,300). An explanatory statement will be provided and written consent to participate will be obtained from all students who participate in surveys, focus groups and interviews.

### Methodological approach

A convergent mixed methods research design [15] will be employed using both quantitative and qualitative data, which will be collected together longitudinally analyzed and interpreted together. Mixed methods has been frequently applied to health education research [16] due to the complexity of learning programs and multiple interactions involved, making it difficult and inaccurate to use a single evaluation method. This mixed methods approach will facilitate evaluation of the various elements and factors that influence student learning and online curriculum delivery by teachers. A framework developed by the same faculty on the factors that contribute to health professions student learning will be used to guide the evaluation. This framework used Contribution Analysis[17] to identify the proximal and distal factors influencing graduate outcomes (Table 1). A range of teacher and student related factors was identified as influencing learning. In addition, the role of WIL in supporting graduate outcomes was highlighted.

**Table 1:** Factors identified as contributing to graduate outcomes

Factors	Description
Teacher dynamic and culture	Previous teaching & industry experience Training & intrinsic interest in teaching Diversity, size & dynamics of the team Health / mental health Policies, procedure & ethical guidelines Teaching performance review Being flexible to students’ learning needs Teaching and contribution to students’ learning experience Being reflexive and willing to change Competing demands outside teaching
Dynamic and culture between teachers, students and WIL educators* * Includes patient as educator • Perceived role and intrinsic interest in teaching • Understanding, skills and support in teaching and assessing • Satisfaction with teaching Learning priorities and challenges in workplace	Teaching logistics, technology and class timetable Diverse learning pace of students Course advisory group inputs Explicit instruction on curricular delivery Strategies to identify struggling students Labor market expectations
Student dynamic and culture	Selection effect, readiness to learn Previous learning experiences and expectations English language proficiency Diversity, size and dynamics of cohort Procedures and guidelines Health / mental health Being reflexive, willing to change, resilience Engaged in learning Competing demands outside learning

The aim of this study is to evaluate the impact of the change to teaching and learning approaches, including online education and changes to work-integrated learning, during the COVID-19 pandemic. More specifically the study will answer the following research questions:

### Student related research questions:

- How have the teaching and learning approaches been received by students?
- What factors (Table 1) have influenced learning outcomes?
- Has there been any influence on learning outcomes, including practical skills and performance on WIL placements?
- How has the change to remote learning impacted upon student academic performance?
- What has been the impact on graduate outcomes, preparedness for practice, work readiness and employability?

### Teacher related research questions:

- What challenges were faced transitioning teaching approaches? What support was provided to enable transition?
- How effectively did teachers adapt to the change and what factors influenced their adaptability?
- Which new modes and approaches to teaching and learning are worth considering for long-term integration?
- What factors (Table 1) have influenced teaching and learning outcomes?

**Fig. 1:**
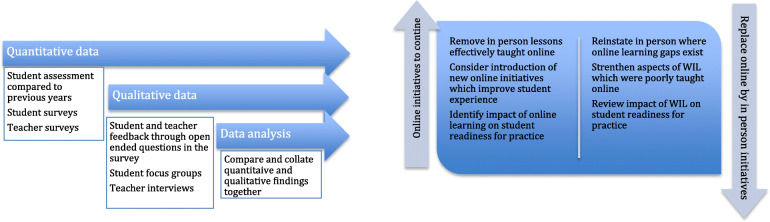
Data collection, analysis and interpretation

An overview of the mixed method design is provided in Fig. 1.

#### Data collection - Quantitative data

##### Assessment data monitoring

Student performance data before and after COVID times will be collected with possible confounders controlled for.

Assessment data will be collected over the duration of the COVID-19 pandemic including the in-semester, work-based and end of the year assessment. The data collected will be compared to similar data from the year 2019 to control for possible confounders. The analysis will provide a direct comparison of student performance in the year prior (when in person learning was present) with the changes to teaching and learning that were introduced during the pandemic (partial/ complete replacement of in person with online learning).

##### Student Evaluation of Teaching and Units (SETU) data

SETU data will be collected from all health professions for all units.

The student evaluation of teaching units provides feedback on student satisfaction with teaching. This data is routinely collected as part of course monitoring and informs teachers if the newly introduced initiatives are well received by students. Similar to the above, current data can be compared to previous years’.

##### Teacher and student surveys

The teacher and student surveys, to be administered, will capture participants’ perspectives of the teaching and learning experienced during the COVID-19 pandemic. Reflecting on the factors contributing to achieving the course learning outcomes (Table 1), students and teachers will be asked to rate each of the factors on a 5-point scale ranging from ‘no influence’ to ‘major influence’.

During the COVID-19 pandemic, both teachers and students have experienced a unique situation. This unique experience may likely yield some uncertainty and stress and require them to adapt and adjust to the new way of being during the pandemic and its unforeseen challenges. Considering this, some of the factors outlined in Table 1 will be examined further in the surveys (e.g. adaptability and resilience). For example, in addition to asking participants about their perceived effectiveness to adapt to changes during the pandemic, they will also be asked to complete a validated nine-item adaptability scale [18] yielding a measure of their adaptability in response to the pandemic.

The surveys will also examine students’ and teachers’ previous experience of, preparedness for, interests in, supports received for, and the challenges encountered in, learning and teaching remotely during the pandemic. The student survey will ask to provide the time students spend typically on learning online and offline including synchronous (e.g. ‘live’ tutorials) and asynchronous activities (e.g. posting to a discussion forum) at the peak of the pandemic. From an economics perspective, the teacher survey will seek to establish an estimate of the total additional hours that teachers have dedicated to transitioning their teaching and assessments to be deliverable over this duration against four categories: (a) considering change (meeting, planning and problem solving); (b) creating change (constructing the required teaching resources or processes, including learning new systems to be able to do so); (c) delivering change (any increase/decrease in teaching delivery time); and (d) supporting change (managing and answering student academic queries and welfare).

In addition, the surveys will include a handful of open-ended questions. For example, the teacher survey will ask participants to describe if they had any experience of providing online/remote education prior to the COVID-19 pandemic and how that experience prepared them for teaching during the pandemic. The student survey will ask participants to elaborate on the reasons for their preference for synchronous and asynchronous learning activities.

Both surveys will ask a range of demographic questions (e.g. sex, cultural and ethnic background, course and year of study/teaching) in order to define characteristics of the sample. The surveys will also collect an expression of interest to participate in longitudinal interviews (teachers) or focus groups (students).

#### Data analysis – quantitative data

Quantitative data relating to student performance, student evaluation of units, quantity of teaching activities and resource utilization will be tested for normality summarized and presented using descriptive statistics. Where appropriate, data will be converted from natural units to financial units (i.e. staff hours converted to salary expenditure). Student demography, unit completion status, grades and student evaluation of units will be analyzed using Analysis of Variance with Bonferroni post-hoc comparison. Differences in these outcomes will be compared across the 2019 and 2020 calendar years using independent t-test. Multivariable logistic regression analysis, adjusting for student demography and period of enrolment, will be used to assess predictors of student performance. Differences in student performance and evaluation of units will be analyzed using inferential statistics including mean differences, ANOVA, linear and logistic regression. A sensitivity analyses will be conducted to explore different permutations of basic assumptions, such as varying the academic level attributed to the staffing hours.

#### Data collection - Qualitative data

##### Longitudinal interviews and focus groups

It is anticipated that the extended duration of the pandemic may have a lasting impact on student learning and confidence in undertaking clinical responsibilities. The lack of clinical exposure for many months at the peak of the pandemic and sustained changes to how students learn (e.g. large group teaching replaced by online learning, minimal peer and teacher interaction occurring in person) may impact students’ learning styles and possibly effect preparedness for future clinical practice. Identifying any barriers to learning early enough may provide an opportunity to institute remediation changes to assist student confidence and competence. Hence, a longitudinal follow up following graduation provides insights if added supervision and support is needed for new graduates completing the course during the pandemic.

To assist in addressing the gaps in learning, an interview and focus group guide was developed [19] with consensus from all researchers. The initial qualitative exploration will focus on experience of teaching and learning during COVID-19 and impact on students and outcomes. A selection of final year students and academic teaching staff who volunteer to participate in the focus groups from all health professional programs, will be invited to participate based on a range of demographics, courses, academic levels etc. (estimated total 50 students and 25 academics for interviews). The members of the research team, with no prior relationship with the student groups, will conduct the focus group and interviews. The students will be reassured of data being de-identified for reporting. A small sample of students and teachers will be selected from the total sample that participate in the initial interviews/focus groups for follow up over time (approximately six students and six teachers).

Follow up interviews will be conducted at the conclusion of the year (graduate point for students) and in 2021 (after graduation). The purpose of the follow up is to explore longer-term impacts on teachers and students. In particular, what have been the permanent changes to teaching practice and for students/graduates, do they feel prepared for practice. These interviews will take a narrative approach asking participants to reflect on experiences of learning and the personal and professional impacts on them. Data will follow graduates from 2020 into their first year of work aiming to capture any impact (positive or negative) COVID-19 has had on their employability or work-readiness.

#### Data analysis - Qualitative data

Data from focus groups and interviews will be transcribed verbatim. Interview data will be analyzed using Ritchie and Spencer’s [20] five-stage framework analysis, which include familiarization, identifying the coding framework, indexing, charting, and mapping and interpretation. We will ensure that we achieve sufficient information power through: [1] focused research questions; the specificity of participants; [3] high quality interview dialogue; [4] large amount of data; and [5] a structured, team-based approach to analysis [21].

In order to ensure trustworthiness in the analysis of qualitative data, we will employ the team-based five-stage framework analysis approach [20] with the use of NVivo. Each stage of the analysis will involve discussion in several rounds of team meetings to compare, contrast and negotiate our interpretations of the data. In addition, we are aware of our positioning in the research through completing a team reflexivity exercise [22] at the beginning of the study. This provide us with a valuable opportunity to understand our diverse background and perspectives that will support more rigorous data interpretation with team members contributing different perspectives and insights into the data collection, analysis and reporting.

### Data analysis - Interpretation

The synthesized findings from each element of the study will be examined side by side by the research team. Comparisons and connections between findings from each data set, including similarities and differences will be identified, discussed and debated [15].

## Discussion

The study aims to evaluate the impact of online education on health science and health professions education in Australia. It is a broad-based faculty-wide evaluation that encompasses the key teaching and learning initiatives introduced in health professions education at the university. Along with being widely implemented over the various courses in biomedical science and health professional education, it also aims to evaluate longitudinal outcome of student learning attributes and impact on graduate outcomes. While there is much being published about the impact of COVID-19 on teaching and learning, this study will be unique in that it will follow the longer-term impacts.

With the introduction of online education delivered over a prolonged duration (up to many months with or without the reintroduction of face-to-face learning), the evaluation will also address how the university workforce engages with a modified curriculum design. It may allow faculty to co-teach units with experienced staff, and hence promote mentorship. Flexible organization of online lessons, with off-site teaching of content may generate a change in curriculum delivery that is worthy of a detailed review. The faculty may be confronted with challenges to create these learning resources; some of them may be quite novel and innovative. It will be valuable to study if the anticipated results are achieved after introducing these variations to course delivery. This can only be achieved through a systematic approach to evaluation, of these educational initiatives during and after the pandemic.

There is a need to assess the learning outcomes achieved through online education. Evidence on feasibility, benefits, shortcomings and modifiable drivers of economic impact of the different types of initiatives that are introduced in online education (and whether they work or not) is needed. If they are found be helpful by learners and teachers, possibly, some of these may be considered for integration in courses in the long term after the pandemic has ceased, and not just be limited to the current situation with enforced distance education [23].

The strengths of this study lie in the large and diverse sample of students, the use of a framework for the selection of variables to examine in detail and the mixed method design that will allow both real and relative concepts to be identified.

## Data Availability

All data and materials can be made available to the journal on request. Request for data can be submitted to the corresponding author by email provided below. arunaz.kumar@monash.edu

## Abbreviations

WIL: Work Integrated Learning
CA: Contribution Analysis
SETU: Student Evaluation of Teaching and Units
